# Removal of migrated metallic prostatic stent by holmium laser

**DOI:** 10.4103/0970-1591.70586

**Published:** 2010

**Authors:** P. M. Mahajan, A. S. Padhye, A. A. Bhave, S. S. Bapat

**Affiliations:** Department of Urology, Ratna Memorial Hospital, Pune (Maharashtra), India

**Keywords:** Holmium laser, prostatic stent

## Abstract

A 90-year-old male with prostatic hyperplasia with a history of ischemic heart disease and right-sided hemiplegia had undergone a Urolume stent placement because of acute urinary retention 9 months earliar. The stent had migrated into the bladder causing dysuria and a poor stream of urine. We fragmented the prostatic stent by Holmium (HO: YAG) laser followed by a laser prostatectomy. After the procedure, the patient voided satisfactorily.

## INTRODUCTION

The permanently implanted super alloy mesh stents have been used for the treatment of urethral stricture and benign prostatic hyperplasia since 1988.[1] Williams, *et al*. were the first to report the use of a permanent (Urolume) stent for the treatment of men with bladder outflow obstruction secondary to the prostate who were considered unfit for surgery.[2] Complications of Urolume stents are migration, encrustation, epithelialization, pain and irritative voiding symptoms that compel removal of the stent.[3]

The HO:YAG laser has been in clinical use in urology for several years but has mainly been used for the treatment of renal stones and benign prostatic hyperplasia. We herein report a case of a patient with benign prostatic hyperplasia (BPH) with a history of ischemic heart disease (IHD) and hemiplegia who had undergone a prostatic stent placement 9 months earlier. We fragmented the stent by Holmium laser followed by a laser prostatectomy.

## CASE REPORT

A 90-year-old male with a history of diabetes mellitus, hypertension, IHD, and right-sided hemiplegia presented to a urologist with recurrent episodes of retention of urine due to prostatic hyperplasia. Considering his medical co-morbidity, a prostatic stent (Urolume) was placed instead of surgery. Approximately 9 months later, he presented to us with severe dysuria, hematuria and poor stream, and urinary tract infection since the last 3 months.

On admission, his physical examination was normal except for right hemiparesis. The prostate had a benign feel on rectal examination. Urinalysis showed pyuria and microscopic hematuria. A urine culture was positive for *E. coli*. An ultrasonography revealed normal kidneys with approximately 75 gms of prostate. An echogenecity of the Urolume stent was visible in the bladder. Hence, removal of stent was planned.

A urethrocystoscopy showed a normal urethra with big median and lateral lobes of prostate. The Urolume stent had migrated into the bladder lying in a pool of pus flakes. We tried to remove it using alligator forceps but the stent could not be withdrawn into the sheath of cystoscope. Hence, the stent was cut into small pieces using a Holmium: YAG laser using 550 micron fiber with a power setting of 1.2 J and 20 HZ. The stent fragments were removed by withdrawing them into the sheath using grasping forceps. Subsequently, the enlarged prostate was enucleated by Holmium laser. The post-operative period was uneventful and the patient could pass urine satisfactorily after removal of the catheter on the second day after the operation.

## DISCUSSION

One of the common complications of prostatic stent is migration into the urinary bladder. Removal of the endoprosthesis may cause trauma to the urethra or external sphincter.[3] The details of the technical aspects of stent removal and its consequences are rare in literature studies. Traditionally, Alligator forceps are used to remove the Urolume stent. Gajewasky and associates removed 43 of 44 stents using this method. Twenty stents were removed intact and 19 were removed wire by wire. An open urethrostomy was required in 1 patient.[4] In our patient, a HO:YAG laser was used to fragment the stent and the stent fragments were removed by withdrawing them into the sheath using grasping forceps.

The HO:YAG laser is currently the workhorse laser in urology since it can be used for multiple soft- and hard-tissue applications. The 2100-nm pulsed wavelength provides the Holmium laser with a unique combination of vaporization and coagulation, allowing a precise cutting action when higher energy levels are applied. A shallow depth of penetration (< 0.5 mm) in water and tissue allows precise energy application and provides a margin of safety. Kural and associates had reported a case in which a HO: YAG laser was used to fragment the endoprosthesis, which led to the easy and uneventful removal of the stent. The power setting was 0.8 J, 6 HZ.[3] In our patient, the stent was cut using a HO: YAG laser with the power setting of 1.2 J, 20 HZ. Higher energy settings were used for rapid fragmentation of the stent. We have not had any complications such as urethral stricture in our patient so far.

There is no specific and standard technique mentioned in the literature for the removal of a Urolume stent as very few studies had addressed this problem. Thus, in difficult cases, the Holmium laser can be an effective tool for this purpose. To our knowledge, we report the third case of removal of a Urolume endoprosthesis using a HO: YAG laser.[3]

## CONCLUSION

The Holmium laser can be a safe and effective option for the removal of a Urolume endoprosthesis.
